# Radioresistance in Prostate Cancer: Focus on the Interplay between NF-κB and SOD

**DOI:** 10.3390/antiox10121925

**Published:** 2021-11-30

**Authors:** Sameera Kumar, Daret St. Clair

**Affiliations:** 1 Department of Radiation Oncology, Fox Chase Cancer Center, Philadelphia, PA 19111, USA; 2 Department of Toxicology and Cancer Biology, University of Kentucky, Lexington, KY 40536, USA; Daret.StClair@uky.edu

**Keywords:** NF-κB, prostate cancer, superoxide dismutase, radiation

## Abstract

Prostate cancer occurs frequently in men and can often lead to death. Many cancers, including prostate cancer, can be initiated by oxidative insult caused by free radicals and reactive oxygen species. The superoxide dismutase family removes the oxygen-derived reactive oxygen species, and increased superoxide dismutase activity can often be protective against prostate cancer. Prostate cancer can be treated in a variety of ways, including surgery, androgen deprivation therapy, radiation therapy, and chemotherapy. The clinical trajectory of prostate cancer varies from patient to patient, but more aggressive tumors often tend to be radioresistant. This is often due to the free-radical and reactive-oxygen-species-neutralizing effects of the superoxide dismutase family. Superoxide dismutase 2, which is especially important in this regard, can be induced by the NF-κB pathway, which is an important mechanism in radioresistance. This information has enabled the development of interventions that manipulate the NF-κB mechanism to treat prostate cancer.

## 1. Introduction

In 2021, almost 250,000 men in the United States are projected to be diagnosed with prostate cancer and almost 34,000 are projected to die from the disease [1]. Numerous cellular mechanisms either contribute to the development of prostate cancer cells or protect against cancer’s development. The variability in the clinical trajectory of prostate cancer reflects its highly heterogeneous nature, with some cases remaining indolent for decades while others rapidly progress to metastatic and treatment refractory stages of the disease.

Treatments for prostate cancer include surgery, radiation, and androgen deprivation, among other therapies. Often these therapies are used in combination with each other. Radiotherapy kills cancer cells by generating free radicals and reactive oxygen species (ROS) that progress to cause fatal cellular and DNA damage. Unfortunately, in some patients, the disease, including castration-resistant prostate cancer and radioresistant prostate cancer, resists therapy.

Mutations in cellular DNA often occur due to insults caused by free radicals and ROS, which can be generated by cellular processes or exogenous causes such as radiation. The superoxide dismutase (SOD) family consists of SOD1, SOD2, and SOD3 proteins that neutralize the first oxygen-derived ROS and provide protection against the development of cancers, including prostate cancer.

Even though SOD can have antitumor effects, it can also contribute to radioresistance, which is often mediated by the NF-κB pathway. One effect of the NF-κB pathway is that transcription of the SOD2 gene increases. In that case, a side effect of neutralizing the ROS is that the cancer cells survive radiotherapy and have prometastasic characteristics.

In this minireview, we review first the basics of prostate cancer and its treatment; then, the role of the NF-κB pathway in SOD induction and radioresistance; next, the role of the SOD family in prostate tissue and prostate cancer; and finally focus on the present translational interventions that utilize the NF-κB pathway and knowledge about SOD.

## 2. Prostate Cancer Treatment and Treatment Resistance

The treatment of prostate cancer is dependent on many factors, including patient performance status, prostate cancer stage, and Gleason score, among others. Treatments include surgery, androgen-deprivation therapy, and radiation therapy for most nonmetastatic cases. Chemotherapy is usually used only for metastatic cases.

Multiple mechanisms are utilized to reduce androgens. Some of the more common options include bilateral orchiectomy, luteinizing hormone-releasing hormone (LHRH) agonists, and antiandrogens. A list of androgen-lowering pharmacologic agents can be found in Table 1. Surgical castration with a bilateral orchiectomy removes both testes, the organs responsible for testosterone production. When LHRH is physiologically secreted by the hypothalamus, the pituitary gland is stimulated and secretes the luteinizing hormone (LH). LH then stimulates the testes and testosterone is produced. This interaction is the reason that an initial androgen surge occurs when LHRH agonists are administered. Over time, due to positive feedback inhibition, the pituitary gland stops producing LH, and thus the testes are not stimulated to produce testosterone. Antiandrogens work by blocking the binding between androgens and the androgen receptor. One of the defining features marking the transition to lethal prostate cancer is the development of castration resistance, defined as chemical and radiographic progression despite castrate (<20 ng/dL) in levels of serum testosterone [2]. This is a complex, multifaceted process involving the interplay between the androgen receptor- signaling axis as well as other molecular pathways that promote tumor growth, progression, and resistance despite suppression of the endogenous ligands testosterone and dihydrotestosterone [3]. More selective, second-generation antiandrogens, such as enzalutamide, have been developed recently. Abiraterone acetate is a newer biosynthesis inhibitor which works to lower the level of androgens by inhibiting CYP17A1, which is found primarily in the gonadal tissues and adrenal glands. Initially, both enzalutamide and abiraterone acetate were approved only for castrate-resistant patients, but recent data show that they also may be beneficial for high-risk, castrate-sensitive patients [4,5].

Radiation therapy, encompassing brachytherapy and external beam radiation, is a commonly utilized curative modality for the treatment of localized prostate cancer, with outcomes comparable to radical prostatectomy [6]. In addition, radiotherapy combined with androgen deprivation therapy improves tumor sensitivity to radiation and disease-specific survival [7,8]. When the disease reoccurs or metastasizes, radioisotopes, including radium-223 (Ra-223) and lutetium-177 (Lu-177), are used for the treatment of prostate cancer. Ra-223 is an alpha-particle emitter with a half-life of 11.4 days. Due to the chemical similarity between calcium and Ra-223, Ra-223 in blood is absorbed by bone. Many bony lesions in metastatic prostate cancer are osteoblastic, and thus Ra-223 is incorporated into these lesions. The alpha particles are then in close proximity to the treatment area. The Phase III ALSYMPCA trial showed a significant overall survival benefit for men with castrate-resistant prostate cancer and at least two bone metastases treated with Ra-223 [9]. Prostate-specific membrane antigen (PSMA) is a transmembrane protein that is overexpressed in prostate cancer [10]. Lu-177 is a beta-particle emitter with a half-life of 6.65 days. It is commonly used for radiopharmaceutical applications. In patients who have high activity on PSMA-specific scans, Lu-177-PSMA-617 is a radioligand which can provide relatively targeted therapy to the PSMA-positive cells, while sparing much of the normal tissue. An open-label, randomized, Phase III trial for metastatic castrate-resistant prostate cancer showed that for men with PSMA-positive radiographic scans, Lu-177-PSMA-617 significantly improved both progression-free survival and overall survival [11].

## 3. Antioxidants and Radioresistance

The generation of ROS is critical for the efficacy of radiation therapy, but it can activate signaling pathways that contribute to radiation resistance and tumor recovery [12]. There are several mechanisms for radioresistance including antiapoptotic, DNA repair, and antioxidant mechanisms, among others. This includes activation and induction of the SOD family.

It is important to note that antioxidant enzymes often do not work alone in the prevention of oxidative damage, but often work in tandem. For example, SOD converts two superoxide radicals (and two hydrogen ions) into hydrogen peroxide and molecular oxygen. Then, other enzymes, such as catalase or peroxidase, further convert two molecules of hydrogen peroxide into water and oxygen. There are several antioxidant enzymes in addition to SOD and catalase. For example, peroxiredoxins (Prx) are an interesting and relatively newly discovered group of enzymes that scavenge peroxide and peroxynitrite. Glutathione, a cellular thiol found in both the cytoplasm and organelles, also reduces ROS and free radicals.

All the antioxidants have been implicated in radioresistance and are topics of active research in varying degrees of emphasis. Peroxiredoxins have a complicated relationship with radioresistance. Prx 2 is significantly upregulated in radioresistant tumors, but Prx 1, 3, 4, 5, and 6 are not [13]. A study that examined radiosensitive and radioresistant esophageal cancer cells found that glutathione levels were preserved in the radioresistant cell line, but the radiosensitive cell line saw a decrease. The authors postulated that this may play a role in radioresistance [14]. Overexpression of catalase, especially mitochondrially localized catalase, is radioprotective as well [15]. Hirose et al. compared SOD2-overexpressing Chinese hamster ovary cells with cells that did not overexpress SOD2, and found that the overexpressing cells had a higher rate of survival after irradiation with gamma rays [16]. A study by Kalen et al. suggested that SOD2 expression increased radioresistance by three- to four-fold [17].

Importantly, molecular pathways governing the transition to castration resistance are often implicated in radiation therapy failure as well [18]. Redox-sensitive transcription factors, such as hypoxia-inducible factor 1-alpha (HIF-1α) and nuclear factor erythroid 2-related factor 2 (Nrf-2) [19,20], have also been found to be radioprotective. HIF-1α is upregulated during hypoxia and contributes to radioresistance by inhibiting apoptosis, repair of radiotherapy-related damage, and several other mechanisms. Nrf-2 both stimulates the transcription of antioxidant molecules and antiapoptotic molecules. A signaling protein known as mTOR utilizes the Nrf2 pathway to upregulate glutathione as a mechanism of radioresistance [21]. In addition to transcription factors such as HIF-1α and Nrf-2, the induction of the NF-κB pathway is a significant mechanism of radioresistance, especially in prostate cancer cells.

## 4. NF-κB Pathway Induction

Exposure to increased oxidative stress is one of the factors which may be implicated in the transformation to more aggressive and castration-resistant prostate cancer [22]. The results of exposure to ROS include alterations of tissue reduction–oxidation balance, which could result in downstream effects such as signal transduction, cell death, and activation of transcription factors; this includes activation of the NF-κB pathway [23,24].

The NF-κB family of proteins includes members p105/p50 (NFκB1), p100/p52 (NFκB2), c-Rel, RelB, and RelA, which are transcription factors that play a critical role in governing cellular processes including growth, survival, and apoptosis [25]. The NF-κB pathway is typically activated by one of two pathways: the canonical or the noncanonical pathway. The members of the NF-κB family combine with each other to form homo- and hetero-dimers. Some, such as p50-RelA heterodimer (in the canonical pathway) and the p52-RelB heterodimer (in the noncanonical pathway), usually act as transcriptional activators, but some homodimers (p50-p50 and p52-p52) can inhibit transcription [26]. In the canonical pathway, p50-RelA is usually inactive in the cytoplasm and is bound to IκB proteins until endogenous signals, such as proinflammatory cytokines, promote its dissociation, which is initiated by the phosphorylation and proteasomal degradation of IκB [27]. One of these proteins (IκBα) is also responsible for binding to the NF-κB heterodimer and causing dissociation from the DNA it is transcribing and sequestration of the NF-κB heterodimer in the cytoplasm [28]. Alternatively, the partial degradation of p100 into p52 in the noncanonical pathway leads to its eventual binding to RelB and to regulate gene transcription after translocation into the nucleus. The canonical and noncanonical pathways regulate different functions and pathways, including those involved in apoptosis, antiapoptosis, cell-cycle regulation, tumor-cell invasion, and angiogenesis [29,30,31,32].

## 5. NF-κB Family and Prostate Cancer

NF-κB is capable of activating over 150 target genes, including inflammatory cytokines, growth factors, and cell adhesion molecules [33]. This activation also causes transcription of the SOD genes. A growing understanding of the far-reaching effects of NF-κB on cell survival has led to efforts to assess its utility as a cancer therapy target.

Increased NF-κB nuclear activity has been found to be associated with progression of prostate cancer in mouse models [34]. In many cancers, tumors that are more dedifferentiated are harbingers of more aggressive disease. Thus, in prostate cancer, tumor cells that have lost their androgen dependence tend to be more aggressive. In prostate cancer, NF-κB expression is upregulated in castration resistance and is predictive of poor prognosis [35,36]. Other studies have shown that androgen insensitivity is associated with NF-κB activation [37,38].

Furthermore, constitutively elevated nuclear levels of RelB are seen in prostate tumors with higher Gleason scores, and correlate with levels of IL-8, a known target gene of the NF-κB pathway and mediator of angiogenesis, which also has other established roles in tumor progression [39]. IL-8 levels have been found to be elevated in metastatic prostate cell lines [40]. In a study by Xu et al., when prostate cancer was irradiated, upregulation of IL-8 made the prostate cancer more resistant to radiation and also caused a decrease in the levels of PSA. Conversely, upregulation of PSA caused decreased radioresistance and decreased levels of IL-8 [41]. Although activation of both the canonical and noncanonical pathways can lead to radioresistance, in a study by Lessard et al., RelB was found in the nucleus of more prostate cancer samples than RelA, p50, and even p52. Only the amount of nuclear RelB correlated to Gleason scores [36].

## 6. Alterations of the NF-κB Pathway That Affect Radioresistance

Because the NF-κB pathway heavily influences radiosensitivity and radioresistance, certain interventions such as gene therapy or pharmacotherapy can alter the pathway and thus make prostate cancer more radiosensitive. It appears that in androgen-dependent prostate cancer, accumulation of p52 was heavily influenced by activation of the androgen receptor. In a study by Lessard et al., when LNCaP cells were exposed to an androgen analog, more nuclear accumulation of p52 occurred. However, when the antiandrogen bicalutamide was used, nuclear p52 level dropped [42]. A study by Fan et al. showed that in mouse epithelial cells, irradiation with 10 cGy induced RelA, and in turn, increased expression of SOD2; several hours later it provided radioprotection against irradiation with 2 Gy. Inhibition of RelA decreased SOD2 and cyclin B1 expression. In a SOD2-knockdown line, radioresistance decreased [43].

Imatinib is a tyrosine kinase inhibitor which is specific for abl, c-kit, and the platelet-derived growth factor receptor. It is typically used to treat leukemia. In response to radiotherapy, RelB moves to the nucleus of the cell where it transcribes genes that will lead to radioresistance. Imatinib decreased this nuclear translocation in androgen-independent PC-3 cells and thus increased radiosensitivity. However, imatinib increased RelB nuclear translocation in androgen-sensitive LNCaP cells [44].

## 7. Role of Superoxide Dismutase in Prostatic Tissue and Prostate Cancer

Free radicals and ROS are generated from mitochondria and other cellular processes, as well as from external sources such as radiation and many pharmacologic agents [45,46]. A common endogenous ROS is H_2_O_2_ [47]. Thus, oxidative stress can be due to endogenous sources or exogenous sources (such as radiotherapy).

Oxidative stress and free radical formation have been linked to the development of several malignancies, including prostate cancer. Antioxidant enzymes, such as those in the SOD family, work to protect the cell from oxidative stress, including damage from free radicals and ROS in intra- and extra-cellular environments. The SOD family contains three isoforms in humans, which include SOD1, SOD2, and SOD3. SOD1 is also known as copper–zinc SOD (CuZnSOD) and is located in the cytosol and mitochondrial intermembrane space. SOD2 is also known as manganese SOD (MnSOD) and is present in the mitochondrial matrix. SOD3 is also known as extracellular SOD (EcSOD). It is only present in some cell types and is the only member of the SOD family present in the extracellular matrix, where it protects the cell from extracellular ROS [48].

A study by Botswick et al. examined PC3 cells, prostatic intraepithelial neoplasia (PIN) tissue, and benign epithelium for SOD1, SOD2, and SOD3 using immunohistochemistry, immunogold electron microscopy, and enzymatic assays [49]. The presence of DNA-damaged adduct 8-hydroxydeoxyguanosine (8-OHdG) was determined in stroma versus epithelium. 8-OHdG, a well-established marker for oxidative DNA damage, frequently has higher levels in cancer tissue [50]. This study found that both PIN and prostate cancer had lower SOD1, SOD2, and catalase than benign tissue, results that support data reported earlier [51,52]. It is possible that lower antioxidant activity leads to decreased protection from oxidative insult. Additionally, evidence of ROS damage with increased 8-OHdG, was found in the epithelium but not the stroma (with no difference among benign epithelium, prostate intraepithelial neoplasia, or prostate cancer), which correlates with prior research [53]. A study from Shima et al. used cDNA microarrays to show that the expression of SOD3 was reduced in prostate cancer compared to normal prostate tissue from the same subjects [54]. In that study, the level of SOD3 expression was not influenced by the grade of the prostate cancer.

Some data support that SOD2 levels are increased in intermediate Gleason scores. A study by Miar et al. looked at levels of SOD2 in prostate, colon, and lung cancers, and found increased levels of either the SOD2 protein, mRNA, or both, for all three cancer types. For prostate cancer, the SOD2 protein, but not the mRNA, increased. Though SOD2 expression was increased in all Gleason scores, this was especially true for Gleason scores 6 and 7 [55]. However, there was no mention of whether these patients had previously received any therapy, which could influence the result. This result is somewhat supported by a study by Quiros-Gonzalez et al., which demonstrated that overexpression of SOD2 in LNCaP cells led to neuroendocrine differentiation, such as expression of synaptophysin, and decreased expression of the androgen receptor [56]. All are indicators of transformation to more aggressive disease.

Interestingly, a study by Venkataraman et al. found that SOD2 expression was reduced in PC-3 cells compared to nonmalignant prostate tissues. When the PC-3 cells were modified to overexpress SOD2, there was a reduction in cell growth rate, an increase in accumulation of extracellular H_2_O_2_, and an increase in the proportion of cells in the G1 phase [57]. The accumulation of hydrogen peroxide may have contributed to the decreased growth rate. ROS contribute to tumorigenesis but also can contribute to tumor cell death [58,59]. Thus, increased levels of H_2_O_2_ can work to reduce proliferation.

Similar results regarding the overexpression of SOD3 were reported by Kim et al. [60]. Inhibition of tumor cell reproduction and migration and invasion of other tissues in PC-3 cells occurred with either SOD3 overexpression or use of recombinant SOD3. SOD3 overexpression also resulted in higher concentrations of H_2_O_2_. There is evidence that cells treated with H_2_O_2_ have higher levels of 8-OHdG expression [61]. Thus, increased concentration of H_2_O_2_ mediated by overexpression of SOD3 could increase 8-OHdG by causing oxidative DNA damage. This could, in turn, result in tumor cell apoptosis. A paper by Chaiswing et al. points out a correlation between SOD3 overexpression and reduced matrix-metalloproteinase activity, and a corresponding reduced tumor invasion in PC-3 cells [62].

SOD3 function can exert an effect opposite to that of the cysteine/glutamate transporter in the control of the redox state of the tumor microenvironment. Prostate cancer with a high Gleason score has a higher cysteine/glutamate transport expression and lower SOD3 expression. This is similar in metastatic prostate cancer. There is less prostate cancer invasion when SOD3 is overexpressed, or the expression of the cysteine/glutamate transporter is decreased. This decreased invasion was accompanied by higher extracellular hydrogen peroxide levels and was reversed by overexpression of catalase, which turned hydrogen peroxide into water and oxygen [63].

## 8. Role of Superoxide Dismutase in Radiotherapy Resistance

As discussed above, SOD2 is thought to protect prostate cells under normal conditions. Because radiation therapy can kill cancer cells by generating free radicals, and because SOD2 is a primary antioxidant enzyme, SOD2 can mitigate the effects of radiotherapy. In the presence of radiation, SOD2 is upregulated by the NF-κB pathway and is radioprotective and antiapoptotic [64,65]. SOD2 induction by RelB is an important mechanism for prostate cancer cells acquiring radioresistance. RelB is a member of the NF-κB family and is a downstream effector of the NF-κB pathway. While RelB and p52 are in the noncanonical pathway, RelB is also regulated by RelA and p50 in the canonical pathway. Cytokines can activate transcription of the SOD2 gene by NF-κB. The SOD2 gene has NF-κB-binding sites which are necessary for this transcription [66,67]. Interestingly, NF-κB binds to an intronic enhancer in the SOD2 gene [68]. A study by Dhar et al. has pointed out the importance of NF-κB as necessary, but not sufficient, for SOD2 induction by cytokines and that nucleophosmin, a nucleolar phosphoprotein, is necessary for the transcription of SOD2 by NF-κB [54].

A study by Josson et al. demonstrated that RelB induced SOD2 in PC-3 cells in response to radiotherapy [69]. This led to increased radioresistance. This was verified by inhibiting RelB with a dominant/negative p100 or specific siRNA, which resulted in significantly lower levels of SOD2 and more radiosensitivity of the PC-3 cells. Similarly, SOD2 has been shown to be upregulated in breast cancer cells as an adaptive response to radiotherapy; this upregulation then confers radiation resistance to additional radiotherapy [64]. Importantly, this radioresistance may be clinically relevant. A study by Margalit et al. found correlations between certain single nucleotide polymorphisms (SNPs) in the SOD2 gene and lethal prostate cancer in a cohort of men who had undergone radiation. These SNPs had no association with lethal prostate cancer in the prostatectomy cohort. Unfortunately, these results were not replicated with a validation cohort [70].

This adds to the literature that supports increased concentrations of SOD in cells does appear to confer radioresistance. Several studies utilized the SOD2 gene in vivo and in vitro to confer radioresistance [71,72,73,74,75]. A more recent study by Zhang et al. used a minicircle plasmid with the SOD2 gene to orally dose mice, which were then irradiated with 31 Gy to the esophagus. The mice that received the SOD2 plasmid had better survival rates than controls. Similarly, mice given the plasmid intravenously had better survival rates with total body irradiation of 9.75 Gy [76]. The study by Josson et al. demonstrated that PC-3 cells (representing high grade prostate cancer) were more radioresistant and had higher nuclear concentrations of RelB than LNCaP cells had (representing low grade prostate cancer) [56]. The study by Josson et al. also demonstrated that SOD2 concentrations were increased for both PC-3 and LNCaP cells in response to radiation, but more superoxide radicals were in the LNCaP cells. When an exogenous SOD2 mimetic was added, the LNCaP cells became more radioresistant. The baseline levels of SOD2 and the activity of the SOD2 were also higher in the PC-3 cell population than in the LNCaP cells. A complex picture results when the results of this study are considered alongside the results of the paper by Venkataraman et al. cited earlier that, showed that PC-3 cells had lower levels of SOD2 compared to immortalized prostate epithelial cells [57]. Together, these studies are consistent with the notion that SOD2 level is reduced when cancer initiates and is increased during progression of the disease.

The radioprotective effect of SOD2 may be limited to oxygen-rich environments. A study from Urano et al. used cDNA to transfect tumor cells with SOD2. Cell lines included a low SOD line, a high SOD line, and two control lines. In the presence of oxygen, both cell lines containing SOD had higher levels of survival after radiotherapy than the control cell lines. In the absence of oxygen, the SOD cell lines were more radiosensitive than the control cell lines. It is important to note that in the absence of oxygen, SOD2 reduced the tumorigenicity of the cell lines containing SOD and did not confer significant radioresistance [77].

Although cells have numerous antioxidant mechanisms, some are more radioprotective than others. A study by Sun et al. created three mammalian cell lines which overexpressed SOD1, SOD2 and glutathione peroxidase, respectively. These cell lines and a control cell line were then irradiated. The cell line which expressed SOD2 provided the most radioprotection followed by the cell line that expressed glutathione peroxidase. Overexpression of SOD1 did not significantly alter the radiosensitivity of the cells [78].

## 9. Early Data on Translational Applications

By utilizing our knowledge about the NF-κB pathway and SOD, clinical interventions could be used to fight prostate cancer. A study by Ismy et al. demonstrated that PC-3 cells undergo more apoptosis after administration of exogenous SOD2 [79]. This occurs via the intrinsic pathway and increased expression of caspase-3, which is known to be an executor caspase for the intrinsic pathway of apoptosis. An increase in the concentration of caspase-3 has been associated with increases in DNA fragmentation and apoptosis [80,81].

Other studies have focused on decreasing the radioresistance of prostate cancer cells. SN52 is a cell-permeable peptide that blocks the noncanonical NF-κB pathway. It works by inhibiting the binding of p52, and thus the translocation of RelB-p52 into the nucleus. SN52 blocks both baseline constitutive SOD2 expression and radiotherapy-induced SOD2 expression. It was found to increase radiosensitivity of prostate cancer cells when given prior to a 2 Gy fraction (a typical daily dose for standard fractionation radiotherapy) [82].

Even previously known substances have proven to be useful to improving radiotherapy. Pretreatment with 1-alpha, 25-dihydroxyvitaminD (3) suppresses increased levels of RelB caused by radiation therapy in prostate cancer cells expressing the vitamin D3 receptor. This in turn causes reduced expression of SOD2 and increased radiosensitivity of prostate cancer cells [83].

There are some clinical results in humans. ATN-224 is an oral SOD1 inhibitor which has also been shown to have a variety of antitumor activities, including antiangiogenic activity, inhibition of epidermal growth factor receptor and platelet-derived growth factor receptors, and prevention of translocation of RelA into the nucleus. A noncomparative Phase II trial administered high-dose and low-dose ATN-224 to patients with biochemically recurrent hormone naive prostate cancer. At six months, 59% and 45% of patients in the low- and high-dose arms, respectively, were biochemically recurrence free [84]. Of course, this needs further study in a larger, comparative study.

## 10. Conclusions

Mutations which result in prostate cancer can originate from ROS-mediated damage. The SOD family is one of the body’s primary antioxidant mechanisms that confers protection from these mutations. Once prostate cancer has developed, SOD level can vary throughout the course of prostate cancer development. Generally, however, the level initially decreases and then gradually increases as the tumor progresses.

Unfortunately, in response to therapy, including radiotherapy, SOD, and especially SOD2, can be upregulated. When it is, it confers radioresistance. This is mediated by the NF-κB pathway, which utilizes both the canonical and the noncanonical pathways. However, the noncanonical pathway has been shown to be particularly significant in the induction of SOD2 in prostate cancer. This knowledge provides the unique opportunity to investigate methods that would block SOD2 in prostate cancer but not in normal tissues. Early, promising, translational developments have been made already that utilize our knowledge of the NF-κB pathway and SOD family to improve the treatment of prostate cancer.

## Figures and Tables

**Table 1:** Androgen pathway modulation therapies used commonly in the treatment of prostate cancer.

Class/Mechanism	Drug	Most Common Uses/Indications in Prostate Cancer
Gonadotropin-releasing Hormone Receptor (GnRH) agonist	Leuprolide acetate	Localized disease (with EBRT) for predetermined time interval Intermittent ADT for biochemical recurrent prostate cancer Continuous for advanced prostate cancer
	Triptorelin pamoate	
	Goserelin acetate	
	Buserelin acetate	
	Histrelin acetate	
GnRH antagonist	Degarelix acetate	Advanced prostate cancer
	Relugolix	Advanced prostate cancer
First-generation androgen receptor antagonists	Bicalutamide	Advanced prostate cancer with GnRH agonist Localized disease with concomitant RT Short-course use to prevent flare effect of GnRH agonist
	Flutamide	Advanced prostate cancer (rarely used)
	Nilutamide	Advanced prostate cancer (rarely used)
Second-generation competitive androgen receptor antagonists (for use with GnRH agonist or antagonist)	Enzalutamide	Metastatic castrate-sensitive prostate cancer Metastatic castrate-resistant prostate cancer
	Apalutamide	Nonmetastatic castrate-resistant prostate cancer Metastatic castrate-sensitive prostate cancer
	Darolutamide	Nonmetastatic castrate-resistant prostate cancer
CYP17 Androgen biosynthesis inhibitor (for use with GnRH agonist or antagonist and must be used with concomitant oral steroid formulation)	Abiraterone acetate	Metastatic castrate-sensitive prostate cancer Metastatic castrate-resistant prostate cancer
